# Association of [^1^H]-MRS quantified liver fat content with glucose metabolism status

**DOI:** 10.1186/s13098-020-00558-8

**Published:** 2020-06-08

**Authors:** Yun-Sheng Wang, Jun Ye, Yong-Hong Cao, Rong Zhang, Xiao-Fang Han, Ling-Ling Zou, Lei Kuang, Ji Zhang, Hu Lian, Jin-Xiang Xia, Qiu Zhang, Wu Dai

**Affiliations:** 1 Department of Endocrinology, The Second People’s Hospital of Hefei, The Affiliated Hefei Hospital of Anhui Medical University, Hefei, 230011 Anhui China; 2 Department of Magnetic Resonance Imaging, The Second People’s Hospital of Hefei, The Affiliated Hefei Hospital of Anhui Medical University, Hefei, 230011 Anhui China; 3 Department of Endocrinology, The First Affiliated Hospital of Anhui Medical University, Hefei, 230022 Anhui China

**Keywords:** Liver fat content, Insulin resistance, Magnetic resonance imaging, T2DM

## Abstract

**Background:**

Previous literatures have implied that the liver fat deposition plays a crucial role in the development and progression of insulin resistance. In the present study, we aimed to investigate the association of liver fat content (LFC) with glucose metabolism status in the population of newly diagnosed type 2 diabetes mellitus (nT2DM), prediabetes mellitus (PDM) and normal controls (NC), and assessing if the LFC could as an indicator for the prediction of T2DM.

**Methods:**

A total of 242 subjects (including 141 nT2DM patients, 48 PDM subjects and 53 NC) were enrolled. The levels of LFC were quantified by using the proton magnetic resonance spectroscopy ([^1^H]-MRS) technique. Clinical and laboratory parameters of study subjects were collected by medical records and biochemical detection. One-way ANOVA or nonparametric test (Kruskal–Wallis) was applied for intergroup comparisons; intergroup comparison was performed in using of Bonferroni multiple-significance-test correction.

**Results:**

There were significantly increased LFC levels in nT2DM (14.72% ± 6.37%) than in PDM (9.62% ± 4.41%) and that of NC groups (5.11% ± 3.66%) (all *p *< 0.001). The prevalence of nonalcoholic fatty liver disease (NAFLD) was also found to be increased in nT2DM (91.48%) than in PDM (85.41%) and that of NC (32.07%) groups. Correlation analysis revealed that the increase of LFC positively associated with fast plasma glucose (FPG), 2 h plasma glucose (PG), Delta G30 and homeostatic model assessment of insulin resistance (HOMA-IR), negatively associated with Delta Ins30, Delta C30, Ins30/G30 _AUC_, CP30/G30 _AUC_, Ins _AUC_/G _AUC_, CP _AUC_/G _AUC_, homeostatic model assessment for β-cell function index (HOMA-β) and matsuda insulin sensitivity index (Matsuda ISI). Multilinear regression analysis showed that LFC, body mass index (BMI) and diastolic blood pressure (DBP) contributed for the prediction of HOMA-IR, and total cholesterol (TC), age, waist circumference (WC) and low-density lipoprotein cholesterol (LDL-C) were the significant contributors for HOMA-β.

**Conclusions:**

Our study revealed an increased LFC level and prevalence of NAFLD in nT2DM than in PDM and that of NC groups, the increase of LFC was closely associated with insulin resistance and impaired glucose metabolism status, may be regarded as potential indicator contributing to the development and progression of T2DM.

## Background

Type 2 diabetes mellitus (T2DM) is a complex, multifactorial, chronic metabolic disease characterized as insulin resistance and impaired pancreatic β-cell function [1, 2]. Up to date, the etiology of T2DM is still not clear. Non-alcoholic fatty liver disease (NAFLD) is defined as the presence of a significant amount of fat deposition in the liver after excluding the secondary causes of fat accumulation in the liver (alcohol consumption, medications or other causes of liver diseases, such as viral hepatitis, autoimmune hepatitis, etc.) [3]. It has been disclosed that NAFLD associated with different types of diseases, such as obesity, diabetes, hypertension and metabolic syndrome [4–7]. The incidence of NAFLD in the general population is approximately 20–30%, but reaches nearly 75% in patients with T2DM [8]. In the past few years, emerging evidence has revealed that the association of NAFLD with an increased risk for T2DM and metabolic syndrome [9, 10].

Liver fat content (LFC) has been regarded as an important clinical indicator for evaluation and diagnosis of NAFLD [11]. Liver biopsy with direct histological visualization remains the current golden standard to evaluate the LFC and diagnose NAFLD. However, due to the invasive nature of the procedure, the clinical application of liver biopsy is limited [12, 13].

The proton magnetic resonance spectroscopy ([^1^H]-MRS) has been recently demonstrated as an accurate, non-invasive option for quantification of LFC [14, 15]. In addition, studies have shown that [^1^H]-MRS had a high consistency with liver biopsy in quantification of LFC, and could be regarded as a reliable and accurate method in assessing LFC [16, 17].

In the present study, we used [^1^H]-MRS to quantify the LFC in nT2DM and PDM and normal controls (NC), investigating the prevalence of NAFLD and exploring the association of LFC with glucose metabolism status and several clinical or laboratory parameters among those groups. In addition, we also evaluate if the LFC could as a reliable and effective indicator for the prediction of T2DM.

## Materials and methods

### Study subjects and methods

This is a single-center, observational study. A total of 242 subjects (141 nT2DM patients, 48 PDM subjects and 53 NC) were recruited from the Department of Endocrinology and medical examination center at the Second People’s Hospital of Hefei, when they first visited the DM clinic. For the clinical diagnosis of PDM and T2DM, the American Diabetes Association diagnostic criteria 2018 was applied [18]. PDM was defined as those without DM but fasting plasma glucose (FPG) value ≥ 5.6 mmol/l and FPG < 6.9 mmol/l or the 2 h plasma glucose (PG) value ≥ 7.8 mmol/l and 2hPG < 11.1 mmol/l after a 75-g oral glucose tolerance test (OGTT) using a glucose load containing the equivalent of 75-g anhydrous glucose dissolved in water. Patients with alcohol consumption, medications or other causes of liver diseases (viral hepatitis, autoimmune hepatitis, Wilson’s disease, hemochromatosis, drug-induced hepatitis) were excluded. NC subjects, without any history of liver or metabolic diseases, were enrolled from the medical examination center. Anthropometric measurement, clinical manifestations and routine laboratory results were obtained from hospital medical records.

The height and weight of each participant clothed in a light gown was measured. Body mass index (BMI) was computed as weight (kg) divided by height (m) squared. Waist circumference (WC) was assessed with a soft tape at the midpoint between the lowest rib margin and iliac crest, and the hip circumference was scaled at the widest level over the greater trochanters. The waist-to-hip ratio (WHR) was calculated as the WC divided by the hip circumference. After a preliminary 5-min rest in the sitting position, blood pressure was measured three times on right arm using an automated sphygmomanometer (OMRON Model HEM-752 FUZZY, Omron Co., Dalian, China), and the average systolic and diastolic blood pressure was calculated.

All study subjects have undergone tests for: total cholesterol (TC), triglyceride (TG), high-density lipoprotein cholesterol (HDL-C), low-density lipoprotein cholesterol (LDL-C), very low-density lipoprotein cholesterol (VLDL-C), alanine aminotransferase (ALT), alkaline phosphatase (ALP), aspartate aminotransferase (AST), γ-glutamyltransferase (GGT), lactate dehydrogenase (LDH), total bilirubin (TBIL), direct bilirubin (DBIL), indirect bilirubin (IBIL), creatinine, uric acid (UA), apolipoprotein-A1 (ApoA1) and apolipoprotein-B (ApoB). The blood biochemical indices were determined by a model 7600 automated bio-analyzer (Hitachi, Tokyo, Japan) or immunoturbidimetry (Roche/Cobas Integra Tinaquant, Roche Diagnostics).

After 10–12 h in the fasting state, the standard 75-g OGTT test was performed in all study subjects (including nT2DM, PDM and NC), then, FPG, 30 min PG, 60 min PG, 120 min (2 h) PG, fasting insulin, 30 min insulin, 60 min insulin, 120 min insulin, fasting C-peptide, 30 min insulin C-peptide, 60 min C-peptide and 120 min insulin C-peptide were measured by the hexokinase method (Audit Diagnostics, Ireland) or the direct chemical luminescence method (Siemens, USA).

The formulas for calculating insulin resistance, β-cell function and insulin sensitivity were as follows: homeostatic model assessment of insulin resistance (HOMA-IR) = fasting serum insulin (mmol/ml) × FPG (mmol/l)/22.5 [19], homeostatic model assessment for β-cell function (HOMR-β) = (fasting insulin (μU/ml) × 20/FPG (mmol/l) − 3.5) [20], and Matsuda insulin sensitivity index (Matsuda ISI) = 10000/(Glu0 × Ins0 × Glumean × Insmean)^1/2^ [21].

### Standard protocol approvals and patient consents

This study was approved by the Ethical Committee of the Second People’s Hospital of Hefei (Hefei, Anhui, China) (approval number 1523). All the study subjects provided informed consent to participate in this study.

### [^1^H]-MRS quantify LFC

All participants underwent the liver ^1^H-MRS to quantify the LFC (GE Signa HDxT 3.0T scanning system, GE Medical Systems, Inc., Waukesha, WI, USA). Sagittal, coronal and axial slices covering the whole liver were preliminarily acquired for positioning of the spectroscopy acquisition voxel. Three independent 20 × 20 × 20 mm voxels were placed within the right lobe of the liver. During the voxel placement, the vessels, bile ducts and focal lesions should be avoided. The proton spectrum was acquired using the body coil after shimming over the volume of interest by means of a point-resolved spectroscopy (PRESS) sequence with the following parameters: repetition time = 3, 333 ms, echo time = 144 ms. The operator calculated the LFC by determining the signal intensity of the in-phase (IP) and out of phase (OP) images at identical locations within regions of interest (ROI). Each ROI was measured three times and the average ROI was calculated as the final value. The captured IP and OP images of the liver were transferred to the GE SAGE software (GE Healthcare Bio-Sciences, Pittsburgh, PA, USA) for further data processing. LFC was calculated as fat fraction 100 × (area under the curve [AUC] fat peak/[AUC fat peak + water peak]) [22–24]. All study subjects were carefully instructed to hold their breath during the end of inspiration to ensure the consistency among subjects. The cut-off value for diagnosis of NAFLD was set as above 5.56% [25].

The repeated measures LFC in using [^1^H]-MRS were independently performed in 100 study subjects within the two different ROI regions by a skilled operator. Variability analysis, by calculating intraclass correlation coefficients (ICC) and depicting Bland–Altman plots, was implemented to evaluate the consistency and reliability of [^1^H]-MRS quantified LFC by the same operator [26]. The results indicated that the repeated quantified LFC by the same operator showed relative high degrees of consistencies (ICC = 0.997) (Additional file 1: Figure S1).

### Statistical analysis

Continuous data were presented as mean ± standard deviation (SD) or the median (interquartile range, IQR) if they were not in normal distribution. One-way ANOVA or nonparametric test (Kruskal–Wallis test) was applied for intergroup comparisons; intragroup comparisons were performed in using Bonferroni multiple-significance-test correction. Chi square test or Fisher’s exact test was used to analyze categorical variables. Statistical correlation analysis was determined by Pearson’s correlation or Spearman’s rank correlation. To identify the contribution of LFC and traditional risk factors on the influence of HOMA-IR or HOMA-β, multivariate linear regression (MLR) analyses were used to detect independent associations of HOMA-IR or HOMA-β with LFC and traditional risk factors of age, BMI, WC, WHR, systolic blood pressure (SBP), diastolic blood pressure (DBP), TC, TG, HDL-C, LDL-C, VLDL-C. Receiver operating characteristic (ROC) analysis was constructed and the area under the curve (AUC) was calculated. Statistical analysis was performed with the use of SPSS software, version 23.0 (SPSS Inc., Chicago, IL, USA). All results with a two tailed *p *< 0.05 were considered to be statistically significant.

## Results

### Characteristics of the study population

Demographic and clinical characteristics of study subjects were displayed in Table 1. There were significant differences in BMI, WC, hip circumference, WHR, SBP, DBP, UA, IBIL, AST, ALT, GGT, LDH, TC, TG, LDL-C, HDL-C, VLDL-C, ApoA1, fasting insulin, FPG, 2hPG, HOMA-IR, HOMA-β and Matsuda ISI among nT2DM, PDM and NC groups (all *p *< 0.05). However, we did not find significant differences in age and gender distributions among those groups (all *p *> 0.05).

**Table 1:** Demographic and clinical characteristic of study subjects

Parameters	nT2DM (n = 141)	PDM (n = 48)	NC (n = 53)	*p* value
Age (year)	50.8 ± 10.1	49.7 ± 13.0	47.0 ± 8.0	0.078
Gender (female/male)	69/72	23/25	25/28	0.974
BMI (kg/cm^2^)	26.5 (25.4, 27.7)	25.7 (24.1, 26.8)	23.6 (22.1, 24.6)	*0.000*
WC (cm)	88.72 ± 9.94	91.87 ± 9.00	83.08 ± 7.81	*0.000*
HIP (cm)	92.06 ± 8.14	98.63 ± 8.39	95.19 ± 6.96	*0.000*
WHR	0.95 (0.92, 1.04)	0.90 (0.88, 0.94)	0.86 (0.83, 0.88)	*0.000*
SBP (mmHg)	135 ± 16	136 ± 16	125 ± 14	*0.001*
DBP (mmHg)	83 ± 11	83 ± 11	77 ± 10	*0.001*
Urea Nitrogen (mmol/l)	5.10 ± 1.48	5.09 ± 1.56	4.82 ± 1.18	0.464
Cr (umol/l)	57.97 ± 14.37	59.98 ± 14.20	62.85 ± 13.80	0.101
UA (umol/l)	342.04 ± 63.36	319.25 ± 75.46	294.15 ± 54.47	*0.000*
TBIL(umol/l)	15.9 (13.2, 21.2)	15.7 (11.5, 20.5)	13.6 (11.3, 16.6)	0.074
DBIL(umol/l)	4.2 (3.3, 5.5)	3.7 (2.7, 5.1)	4.2 (3.0, 5.1)	0.540
IBIL(umol/l)	11.9 (9.6, 16.3)	12.0 (8.4, 15.1)	9.6 (7.9, 12.5)	*0.045*
ALP (U/l)	76 ± 21	76 ± 21	71 ± 22	0.289
AST (U/l)	20 (16, 28)	24 (18, 29)	19 (16, 24)	*0.019*
ALT (U/l)	35 (32, 38)	31 (26, 36)	18 (13, 27)	*0.000*
GGT (U/l)	36 (26, 54)	34 (19, 68)	23 (16, 34)	*0.007*
LDH (U/l)	166 ± 33	189 ± 38	184 ± 32	*0.000*
TG (mmol/l)	2.4 ± 0.6	1.9 ± 0.7	1.4 ± 0.6	*0.000*
TC (mmol/l)	5.4 ± 0.3	4.8 ± 0.2	4.1 ± 0.4	*0.000*
HDL-C (mmol/l)	1.4 ± 0.3	1.5 ± 0.3	1.6 ± 0.4	*0.000*
LDL-C (mmol/l)	3.3 ± 0.4	2.9 ± 0.7	2.2 ± 0.2	*0.000*
VLDL-C (mmol/l)	0.4 (0.3, 0.6)	0.3 (0.2, 0.5)	0.2 (0.1, 0.4)	*0.003*
ApoB (g/l)	0.9 (0.7, 1.1)	0.9 (0.7, 1.0)	0.9 (0.8, 1.0)	0.403
ApoA1 (g/l)	1.1 ± 0.2	1.2 ± 0.2	1.2 ± 0.2	*0.000*
FPG (mmol/l)	7.44 ± 1.07	5.54 ± 0.65	4.84 ± 0.62	*0.000*
2hPG (mmol/l)	16.90 (14.00, 17.88)	8.93 (8.05, 9.75)	6.05 (5.34, 6.71)	*0.000*
Fasting Insulin (mU/l)	6.31 (5.24, 8.47)	7.77 (6.05, 8.70)	6.63 (5.54, 8.26)	*0.021*
HOMA-IR	2.20 (1.73, 2.78)	1.82 (1.45, 2.22)	1.44 (1.19, 1.86)	*0.000*
HOMA-β	32.89 (24.32, 45.60)	81.09 (59.16, 109.50)	117.11 (74.55, 158.77)	*0.000*
Matsuda ISI	93.38 ± 38.31	78.76 ± 24.14	114.64 ± 44.28	*0.000*

*ApoA1* Apolipoprotein A1, *ApoB* Apolipoprotein B, *ALP* alkaline phosphatase, *AST* aspartate aminotransferase, *ALT* alanine transaminase, *BMI* body mass index, *Cr* creatine, *DBIL* direct bilirubin, *DBP* diastolic blood pressure, *FPG* fasting plasma glucose, *GGT* γ-glutamyltransferase, *HOMA-IR* homeostatic model assessment of insulin resistance, *HOMA-β* homeostatic model assessment for β-cell function, *HDL-C* high-density lipoprotein cholesterol, *IBIL* indirect bilirubin, *LDH* lactate dehydrogenase, *LDL-C* low-density lipoprotein cholesterol, *nT2DM* newly diagnosed type 2 diabetes mellitus, *NC* normal control, *PDM* prediabetes mellitus, *SBP* systolic blood pressure, *TC* total cholesterol, *TG* triglycerides, *TBIL* total bilirubin, *UA* uric acid, *VLDL-C* very low-density lipoprotein cholesterol, *WC* waist circumference, *WHR* waist-to-hip ratio

### Comparisons and distribution of LFC among study groups

**Fig. 1:**
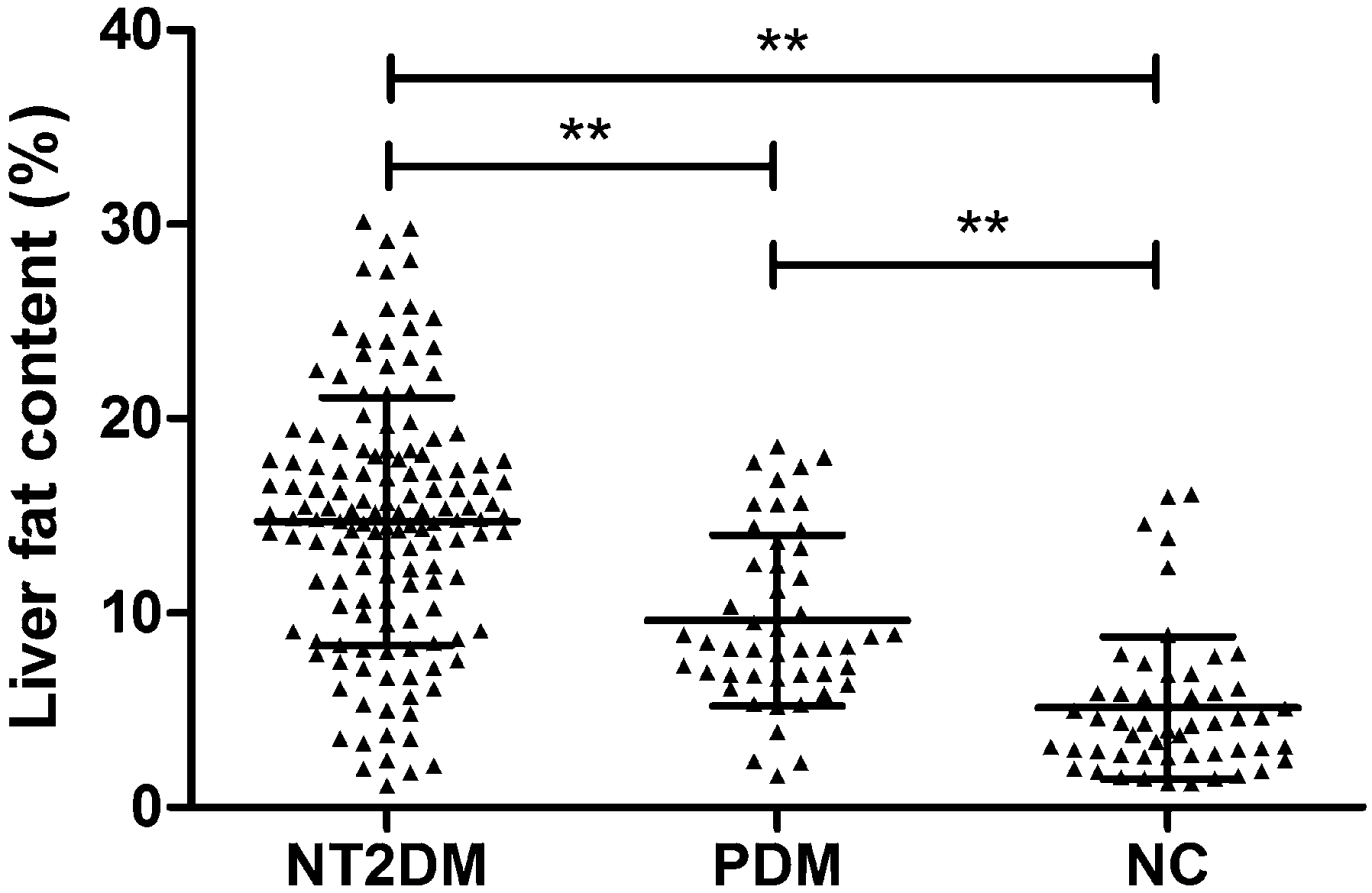
The comparison of LFC in nT2DM, PDM and NC groups. *LFC* liver fat content, *nT2DM* newly diagnosed type 2 diabetes mellitus, *PDM* pre-diabetes mellitus, *NC* normal controls

There was a significant difference of LFC level among three groups (all *p *< 0.05) (Fig. 1). In compared to NC group (5.11% ± 3.66%), a significantly increased LFC level was observed in nT2DM (14.72% ± 6.37%) and PDM groups (9.62% ± 4.41%) (both *p *< 0.05). Furthermore, a significantly higher LFC level was found in nT2DM group than in PDM group (14.72% ± 6.37% vs 9.62% ± 4.41%) (*p *< 0.05).

**Fig. 2:**
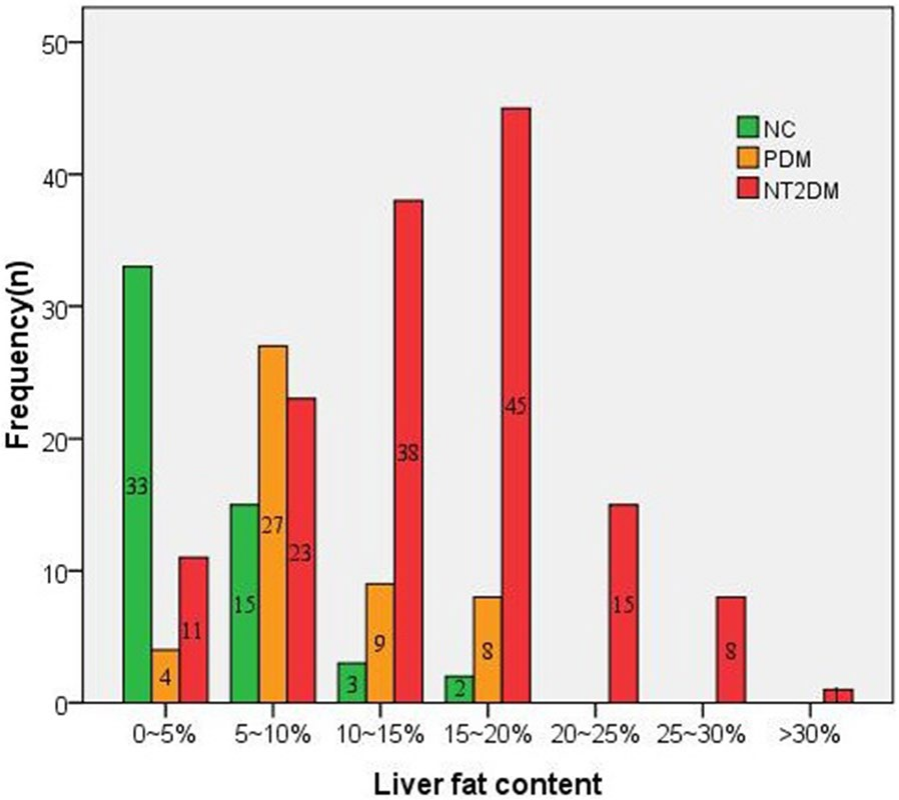
The frequency distribution of LFC in nT2DM, PDM and NC groups. *LFC* liver fat content, *nT2DM* newly diagnosed type 2 diabetes mellitus, *PDM* pre-diabetes mellitus, *NC* normal controls

The frequency distribution of LFC among nT2DM, PDM and NC groups was shown in Fig. 2. We could observe that the frequency of LFC among NC, PDM and nT2DM groups mainly distributed in LFC of 0–5% (68.75%), LFC of 5–10% (41.54%) and LFC of 10–20% (53.81%), respectively. In addition, the detection of NAFLD among nT2DM, PDM and NC groups were 91.48%, 85.41% and 32.07%, respectively.

### Correlation analysis of LFC with clinical and laboratory parameters among study groups

Univariate correlation analysis revealed that LFC was positively correlated with BMI, WHR, SBP, DBP, FPG, HOMA-IR and negatively correlated with TBIL, DBIL and IBIL in nT2DM group (all *p *< 0.05). In PDM group, there was a significantly positive association of LFC with BMI, FPG, UA and HOMA-IR (all *p *< 0.05). Moreover, in NC group, LFC showed a positively association with FPG, urea nitrogen, ALT, ALP and HOMA-IR, and a negatively association with ApoA1 (all *p *< 0.05). However, no significant correlations of LFC with other clinical and quantitative laboratory parameters among those three groups were observed (all *p *> 0.05) (Table 2).

**Table 2:** Correlation coefficients between LFC with demographic and laboratory parameters

Parameters	nT2DM (n = 141)		PDM (n = 48)		Normal Control (n = 53)	
	r	*p*	r	*p*	r	*p*
Age	− 0.024	0.781	0.072	0.626	0.151	0.280
BMI	0.268	*0.001*	0.416	*0.003*	0.223	0.108
WHR	0.314	*0.000*	0.183	0.214	0.107	0.446
SBP	0.190	*0.024*	0.256	0.079	0.223	0.109
DBP	0.178	*0.035*	0.074	0.616	0.114	0.414
FPG	0.222	*0.008*	0.385	*0.007*	0.398	*0.003*
2hPG	0.096	0.258	0.110	0.458	0.094	0.505
Urea Nitrogen	− 0.042	0.617	0.227	0.121	0.439	*0.001*
Cr	− 0.071	0.403	0.215	0.142	0.036	0.800
UA	0.121	0.151	0.390	*0.006*	0.178	0.203
TBIL	− 0.244	*0.004*	0.022	0.880	− 0.186	0.183
DBIL	− 0.213	*0.011*	0.099	0.502	− 0.267	0.053
IBIL	− 0.225	*0.007*	0.005	0.974	− 0.040	0.774
ALT	0.119	0.161	− 0.030	0.840	0.304	*0.027*
AST	0.060	0.483	0.038	0.800	0.140	0.316
GGT	0.014	0.867	0.122	0.408	0.135	0.336
ALP	0.038	0.653	− 0.009	0.951	0.291	*0.035*
LDH	0.062	0.469	− 0.033	0.823	0.015	0.915
TG	− 0.059	0.486	− 0.109	0.461	0.195	0.162
TC	− 0.042	0.621	− 0.056	0.705	0.186	0.182
HDL-C	− 0.038	0.657	− 0.157	0.286	− 0.262	0.058
LDL-C	− 0.008	0.922	− 0.041	0.780	0.192	0.169
VLDL-C	0.119	0.161	− 0.077	0.602	0.219	0.116
ApoB	− 0.112	0.185	− 0.163	0.267	0.023	0.868
ApoA1	− 0.008	0.922	− 0.193	0.189	− 0.289	*0.036*
HOMA-IR	0.262	*0.002*	0.400	*0.005*	0.274	*0.047*
HOMA-β	− 0.014	0.873	− 0.073	0.621	− 0.221	0.112
Matsuda ISI	− 0.149	0.078	− 0.056	0.706	− 0.234	0.092

*ApoA1* Apolipoprotein A1, *ApoB* Apolipoprotein B, *ALP* alkaline phosphatase, *AST* aspartate aminotransferase, *ALT* alanine transaminase, *BMI* body mass index, *Cr* creatine, *DBIL* direct bilirubin, *DBP* diastolic blood pressure, *FPG* fasting plasma glucose, *GGT* γ-glutamyltransferase, *HOMA-IR* homeostatic model assessment of insulin resistance, *HOMA-β* homeostatic model assessment for β-cell function, *HDL-C* high-density lipoprotein cholesterol, *IBIL* indirect bilirubin, *LFC* liver fat content, *LDH* lactate dehydrogenase, *LDL-C* low-density lipoprotein cholesterol, *nT2DM* newly diagnosed type 2 diabetes mellitus, *NC* normal control, *PDM* prediabetes mellitus, *SBP* systolic blood pressure, *TC* total cholesterol, *TG* triglycerides, *TBIL* total bilirubin, *UA* uric acid, *VLDL-C* very low-density lipoprotein cholesterol, *WC* waist circumference, *WHR* waist-to-hip ratio

### Comparisons of several glucose metabolism indicators among LFC quartile groups

Given the distribution of LFC among study subjects, we calculated the quartile of LFC, a quartile divides LFC into three points (a lower quartile, median, and upper quartile) to inform four groups of the LFC (Q1: LFC < 5.89%, Q2: 5.89% ≤ LFC < 11.62%, Q3: 11.62% ≤ LFC < 16.26% and Q4: LFC ≥ 16.26%).

The results indicated significant differences of FPG, 2hPG, Delta G30, Delta Ins30, Delta C30, Ins30/G30 _AUC_, CP30/G30 _AUC_, Ins _AUC_/G _AUC_, CP _AUC_/G _AUC_, HOMA-IR, HOMA-β and Matsuda ISI among four groups (all *p *< 0.05) (Table 3). With the increase of LFC, the FPG, 2hPG and HOMA-IR showed a comparable increase. Nevertheless, Matsuda ISI, Delta Ins30, Delta C30, Ins30/G30 _AUC_, CP30/G30 _AUC_, Ins _AUC_/G _AUC_, CP _AUC_/G _AUC_ and HOMA-β showed a tendency of decrease. Delta G30 exerted an increased trend from Q1 to Q3, then showed a descent trend in Q4.

**Table 3:** Comparison of insulin resistance and β-cell function among groups divided by LFC quartile

Parameters	LFC < 5.89% (n = 61)	5.89% ≤ LFC < 11.62% (n = 61)	11.62% ≤ LFC < 16.26% (n = 60)	16.26% ≤ LFC (n = 60)	*p* value
FPG (mmol/l)	5.26 ± 1.00	6.28 ± 1.35^a^	6.99 ± 1.36^bd^	7.45 ± 1.09^ce^	*0.000*
2hPG (mmol/l)	6.70 (5.73, 9.59)	10.42 (8.01, 17.16)^a^	15.23 (10.49, 17.68)^b^	16.89 (12.90, 17.86)^ce^	*0.000*
Delta G30	4.23 ± 1.41	5.52 ± 1.59^a^	5.60 ± 1.58^b^	5.51 ± 1.58^c^	*0.000*
Delta Ins30	39.96 (18.02, 56.95)	24.03 (12.28, 47.62)	13.70 (5.87, 31.97)^b^	10.60 (4.67, 23.61)^ce^	*0.000*
Delta C30	2.69 (1.80, 4.27)	2.09 (1.10, 3.79)	1.40 (0.63, 2.80)^b^	1.05 (0.71, 1.78)^ce^	*0.000*
Ins30/G30 _AUC_	9.26 (4.49, 14.24)	4.49 (2.17, 9.55)^a^	2.36 (1.01, 6.39)^b^	2.11 (0.84, 4.08)^ce^	*0.000*
CP30/G30 _AUC_	0.65 (0.38, 1.06)	0.38 (0.19, 0.81)^a^	0.28 (0.12, 0.52)^b^	0.21 (0.11, 0.33)^ce^	*0.000*
Ins _AUC_/G _AUC_	5.52 (3.52, 7.79)	3.20 (1.33, 5.24)^a^	1.86 (1.20, 4.50)^b^	1.51 (1.04, 3.56)^ce^	*0.000*
CP _AUC_/G _AUC_	0.57 (0.38, 0.85)	0.44 (0.22, 0.70)	0.31 (0.19, 0.55)^b^	0.29 (0.17, 0.43)^c^	*0.000*
HOMA-IR	1.62 ± 0.58	1.98 ± 0.76	2.31 ± 0.78^b^	2.64 ± 1.14^ce^	*0.000*
HOMA-β	93.38 (56.90, 141.29)	55.61 (32.92, 94.99)^a^	40.48 (25.21, 78.90)^b^	34.74 (26.58, 56.84)^c^	*0.000*
Matsuda ISI	110.23 ± 42.91	95.46 ± 38.55	88.92 ± 34.31^b^	85.69 ± 36.26^c^	*0.005*

*AUC* area under the curve, *PG* plasma glucose, *FPG* fasting plasma glucose, *HOMA-IR* homeostatic model assessment of insulin resistance, *HOMR-β* homeostatic model assessment for β-cell function, *LFC* liver fat content, *Matsuda ISI* Matsuda insulin sensitivity index

^a^Significant difference in LFC < 5.89% versus 5.89% ≤ LFC < 11.62

^b^Significant difference in LFC < 5.89% versus 11.62% ≤ LFC < 16.26%

^c^Significant difference in LFC < 5.89% versus 16.26% ≤ LFC

^d^Significant difference in 5.89% ≤ LFC < 11.62 versus 11.62% ≤ LFC < 16.26%

^e^Significant difference in 5.89% ≤ LFC < 11.62 versus 16.26% ≤ LFC

^f^Significant difference in 11.62% ≤ LFC < 16.26% versus 16.26% ≤ LFC

### Correlation analysis of LFC with several glucose metabolism indicators

Univariate correlation analysis indicated that LFC was positively correlated with FPG, 2hPG, Delta G30 and HOMA-IR, negatively correlated with Delta Ins30, Delta C30, Ins30/G30 _AUC_, CP30/G30 _AUC_, Ins _AUC_/G _AUC_, CP _AUC_/G _AUC_, HOMA-β and Matsuda ISI (all *p *< 0.05). (Additional file 2: Table S1).

### MLR to identify the contributors for HOMA-IR and HOMA-β

First, HOMA-IR was set as dependent variable, independent variables of LFC and traditional risk factors (age, gender, BMI, WC, SBP, DBP, TC, TG, HDL-C, LDL-C and VLDL-C) were included in MLR model, the results indicated that BMI, LFC and DBP were the significant contributors that closely associated with HOMA-IR (Additional file 3: Table S2).

Second, we also analyzed the contribution of LFC and traditional risk factors (age, gender, BMI, WC, SBP, DBP, TC, TG, HDL-C, LDL-C, VLDL-C) on HOMA-β, the MLR model suggested that TC, age, WC, LDL-C were the significant contributors for HOMA-β (Additional file 3: Table S2).

## Discussion

Although previous studies showed that LFC may be closely associated with several clinical and laboratory parameters like BMI or HOMA-IR, however, limited study has investigated the LFC and its relationship with clinical and laboratory parameters in nT2DM and PDM. In the present study, we have used [^1^H]-MRS to measure the LFC among nT2DM, PDM and NC groups, and the results revealed that there was an increased LFC level and detection rate of NAFLD in patients with nT2DM than in PDM and those of NC. LFC was shown to be positively associated with FPG and HOMA-IR in all three groups. In addition, we found that there were significant differences of several glucose metabolism indicators among four LFC quartile groups; from Q1 to Q4, the levels of FPG, 2hPG and HOMA-IR showed a comparable increase, however, Matsuda ISI, Delta Ins30, Delta C30, Ins30/G30 _AUC_, CP30/G30 _AUC_, Ins _AUC_/G _AUC_, CP _AUC_/G _AUC_ and HOMA-β showed a decedent trend. Correlation analysis also supported a positive correlation of LFC with FPG, 2hPG and HOMA-IR, and a negatively correlation of LFC with Matsuda ISI, Delta Ins30, Delta C30, Ins30/G30 _AUC_, CP30/G30 _AUC_, Ins _AUC_/G _AUC_, CP _AUC_/G _AUC_ and HOMA-β. It has been demonstrated that increasing accumulation of intrahepatic triglyceride (IHTG) was associated with a step-wise increase in plasma fasting insulin levels and continuous reduction in hepatic insulin extraction, however, the level of FPG showed no association with the increase of IHTG [27]. Given that HOMA-IR was mainly driven by plasma insulin levels, HOMA-IR levels increased with worsening IHTG accumulation. Furthermore, the possibility therefore arises that the relationship between hepatic steatosis and insulin resistance is a vicious cycle, in which systemic insulin resistance leads to hepatic steatosis, and hepatic steatosis then leads to an exacerbation of hepatic insulin resistance.

There is a widely held perception that liver steatosis is associated with increased production of insulin from the beta cell in order to compensate for whole-body insulin resistance, insulin resistance is not thought to influence beta cell function per se, it just leads to more insulin being produced. Study has suggested that, in apparently healthy older adults, liver steatosis is associated with reduced hepatic insulin extraction and beta cell dysfunction after adjusting confounding factors of age, sex and alcohol consumption [28]. In our study, there are several explanations that may cause the decreased trend of HOMA-β. First, the time of newly diagnosis T2DM and PDM patient’s recruitment fall behind the disease onset, thus, may cause the different status on impaired β-cell function. Second, the toxicity of lipid could impair the pancreatic function and decrease the insulin compensatory secretion, and lead to a decrease of HOMA-β. In addition, the study sample size of among study groups is differed, the relatively small sample size of PDM and NC groups may also cause the decrease of HOMA-β. Furthermore, although we did not quantify pancreatic fat content in the present study, the accumulation of ectopic fat in the pancreas is increasingly recognized as a cause of beta-cell dysfunction.

MLR analysis indicated that, LFC and traditional risk factors of BMI and DBP represented the significant contributors for the presence of HOMA-IR. BMI has been demonstrated as the marker for evaluation of overweight or obesity, and was also considered to be the strongest influencing factor for the peripheral insulin resistance [29]. HOMA-IR mainly reflects insulin sensitivity in fasting state, that is, the degree to which insulin inhibits liver sugar output, and also the severity of liver insulin resistance. Although BMI reflects an individual’s overall obesity and associated with blood pressure, it does not accurately reflect the extent to which fat is deposited in organs. Therefore, compared with other traditional factors, LFC can accurately and truly assess the extent of fat heterotopic deposition and more directly reflect insulin resistance in liver.

As for the MLR analysis of HOMA-β, the results revealed that TC, age, WC and LDL-C were the greater contributor associated with HOMA-β. The potential influence of TC and LDL-C on HOMA-β may be attributed to the inhibited pancreatic function caused by the toxicity of lipid [30]. In addition, the pancreatic function gradually declined with the increase of age, and then affects the HOMA-β. Increase of WC has been demonstrated to be associated with increased HOMA-IR and decreased insulin sensitivity, thus could lead to insulin compensatory secretion and impair pancreatic function.

Our results revealed an association between LFC and glucose metabolism status, where the excessive accumulation of liver fat strongly correlated with insulin resistance, impaired insulin secretive function and abnormality of glucose metabolism. It remains always controversial whether fat deposition in the liver is a cause or consequence of insulin resistance. Some investigators have illustrated that liver fat accumulation closely associated with BMI, LDL, TG, insulin resistance and FPG, suggesting that ectopic fat accumulation in the liver affects the normal metabolism of lipids and may contribute to the development and progression of diabetes [28, 31, 32]. However, several observations indicated that the intrahepatic alterations in glucose and fat metabolism could also cause liver steatosis, and the liver fat accumulation does not seem to be sufficient or necessary to induce hepatic insulin resistance [33–35].

There are some shortcomings in the present study that need to be acknowledged. First, this study is an observational study with a case–control design that could not prove the causal relationship due to the lack of clear time logic. Second, the selection of study sample is based on single hospital, and may have selection bias. Third, [^1^H]-MRS is time consuming to perform and can depict the fat content of only a portion of the organs; the placement of voxels requires operator expertise, especially in small organs of irregular shape, thus the accuracy of MRS can be compromised. Furthermore, due to a relatively small sample size, especially in PDM and NC, it may impair the reliability of our results. Hence, further community-based studies with a large sample size are still required to confirm our results.

## Conclusions

In summary, our study has indicated that the increase of LFC plays an important role in insulin resistance, abnormal glucose metabolism status and eventually diabetes, and may be regarded as potential indicators for abnormal glucose tolerance and T2DM. Early intervention, ideally as soon as abnormalities in LFC are detected, is of great importance for the prevention of T2DM.

## Supplementary information

**Additional file 1: Figure S1.** Bland–Altman analysis for intra-session repeatability for [^1^H]-MRS defined LFC in two ROI regions of 100 subjects.

**Additional file 2: Table S1.** Correlation of LFC with parameters regarding insulin resistance and β-cell function.

**Additional file 3: Table S2.** Multilinear regression analysis of HOMA-IR and HOMA-β with different predictors.

## Data Availability

The data and material that support the findings of this study are available from the corresponding author upon reasonable request.

## Abbreviations

AUC: Area under the curve
ALT: Alanine aminotransferase
ALP: Alkaline phosphatase
AST: Aspartate aminotransferase
ApoA1: Apolipoprotein A1
ApoB: Apolipoprotein B
BMI: Body mass index
DBP: Diastolic blood pressure
DBIL: Direct bilirubin
FPG: Fasting plasma glucose
GGT: γ-Glutamyltransferase
HDL-C: High-density lipoprotein cholesterol
[^1^H]-MRS: Proton magnetic resonance spectroscopy
HOMA-IR: Homeostatic model assessment of insulin resistance
HOMR-β: Homeostatic model assessment for β-cell function
IP: In-phase
ICC: Intraclass correlation coefficients
IBIL: Indirect bilirubin
IQR: Interquartile range
LDL-C: Low-density lipoprotein cholesterol
LDH: Lactate dehydrogenase
LFC: Liver fat content
Matsuda ISI: Matsuda insulin sensitivity index
MLR: Multivariate linear regression
NAFLD: Non-alcoholic fatty liver disease
NC: Normal controls
nT2DM: Newly diagnosed T2DM
OGTT: Oral glucose tolerance test
OP: Out of phase
PDM: Prediabetes mellitus
PG: Plasma glucose
PRESS: Point-resolved spectroscopy
ROC: Receiver operating characteristic
ROI: Regions of interest
SD: Standard deviation
SBP: Systolic blood pressure
TBIL: Total bilirubin
TC: Total cholesterol
TG: Triglyceride
T2DM: Type 2 diabetes mellitus
UA: Uric acid
VLDL-C: Very low-density lipoprotein cholesterol
WC: Waist circumference
WHR: Waist-to-hip ratio
