# Supplementary figures and images for: Association of [1H]-MRS quantified liver fat content with glucose metabolism status
Source: Diabetol Metab Syndr. 2020 Jun 8;12:51. doi: 10.1186/s13098-020-00558-8 (PMC7282165; doi:10.1186/s13098-020-00558-8)

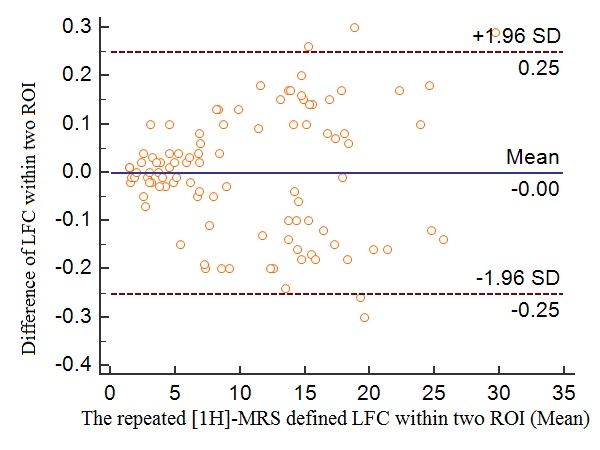

Supplement: Supplementary file 1 — Additional file 1: Figure S1. Bland–Altman analysis for intra-session repeatability for [1H]-MRS defined LFC in two ROI regions of 100 subjects. [file 13098_2020_558_MOESM1_ESM.jpg]
