# Supplementary material for: Association of [1H]-MRS quantified liver fat content with glucose metabolism status
Source: Diabetol Metab Syndr. 2020 Jun 8;12:51. doi: 10.1186/s13098-020-00558-8 (PMC7282165; doi:10.1186/s13098-020-00558-8)
Supplement: Supplementary file 2 — Additional file 2: Table S1. Correlation of LFC with parameters regarding insulin resistance and β-cell function. [file 13098_2020_558_MOESM2_ESM.docx]

Table S1 Correlation of LFC with parameters regarding insulin resistance and β-cell function

| Parameters | LFC | |
| --- | --- | --- |
|  | r | *p* |
| FPG | 0.597 | **0.000** |
| 2hPG | 0.562 | **0.000** |
| Delta G30 | 0.281 | **0.000** |
| Delta Ins30 | -0.390 | **0.000** |
| Delta C30 | -0.350 | **0.000** |
| Ins30/G30 _AUC_ | -0.425 | **0.000** |
| CP30/G30 _AUC_ | -0.404 | **0.000** |
| Ins _AUC_/G _AUC_ | -0.432 | **0.000** |
| CP _AUC_ /G _AUC_ | -0.351 | **0.000** |
| HOMA-IR | 0.500 | **0.000** |
| HOMA-β | -0.429 | **0.000** |
| Matsuda ISI | -0.214 | **0.001** |

AUC: area under the curve; PG: plasma glucose; FPG: fasting plasma glucose; HOMA-IR: homeostatic model assessment of insulin resistance; HOMR-β: homeostatic model assessment for β-cell function; LFC: liver fat content; Matsuda ISI: Matsuda insulin sensitivity index
