# Supplementary material for: Association of [1H]-MRS quantified liver fat content with glucose metabolism status
Source: Diabetol Metab Syndr. 2020 Jun 8;12:51. doi: 10.1186/s13098-020-00558-8 (PMC7282165; doi:10.1186/s13098-020-00558-8)
Supplement: Supplementary file 3 — Additional file 3: Table S2. Multilinear regression analysis of HOMA-IR and HOMA-β with different predictors. [file 13098_2020_558_MOESM3_ESM.docx]

Table S2 Multilinear regression analysis of HOMA-IR and HOMA-β with different predictors.

| Parameters | Model | R | R^2^ | Adjusted R^2^ | Standard Error of the Estimate | Model *p* value | Predictors | β | 95%CI (β) | VIF |
| --- | --- | --- | --- | --- | --- | --- | --- | --- | --- | --- |
| HOMA-IR | Model 1 | 0.466 | 0.217 | 0.214 | 0.14112 | 0.000 | Constant | -1.822 | -2.783, -0.860 | - |
|  |  |  |  |  |  |  | BMI | 0.153 | 0.116, 0.191 | 1.000 |
|  | Model 2 | 0.520 | 0.271 | 0.265 | 0.13516 | 0.000 | Constant | -1.170 | -2.148, -0.191 | - |
|  |  |  |  |  |  |  | BMI | 0.112 | 0.071, 0.153 | 1.292 |
|  |  |  |  |  |  |  | LFC | 0.036 | 0.019, 0.052 | 1.292 |
|  | Model 3 | 0.543 | 0.295 | 0.286 | 0.13303 | 0.000 | Constant | -1.927 | -3.027, -0.828 | - |
|  |  |  |  |  |  |  | BMI | 0.099 | 0.058, 0.141 | 1.353 |
|  |  |  |  |  |  |  | LFC | 0.033 | 0.016, 0.050 | 1.310 |
|  |  |  |  |  |  |  | DBP | 0.014 | 0.004, 0.023 | 1.112 |
| HOMA-β | Model 1 | 0.466 | 0.217 | 0.214 | 0.26592 | 0.000 | Constant | 351.67 | 284.182, 419.158 | - |
|  |  |  |  |  |  |  | TC | -55.892 | -69.380, -42.405 | 1.000 |
|  | Model 2 | 0.505 | 0.255 | 0.249 | 0.26187 | 0.000 | Constant | 405.359 | 332.690, 478.028 | - |
|  |  |  |  |  |  |  | TC | -53.367 | -66.632, -40.102 | 1.012 |
|  |  |  |  |  |  |  | Age | -1.331 | -2.086, -0.576 | 1.012 |
|  | Model 3 | 0.524 | 0.275 | 0.266 | 0.25931 | 0.000 | Constant | 257.293 | 122.376, 392.210 | - |
|  |  |  |  |  |  |  | TC | -49.691 | -63.108, -36.273 | 1.058 |
|  |  |  |  |  |  |  | Age | -1.276 | -2.023, -0.528 | 1.015 |
|  |  |  |  |  |  |  | WC | 1.360 | 0.311, 2.409 | 1.053 |
|  | Model 4 | 0.541 | 0.293 | 0.281 | 0.25679 | 0.000 | Constant | 247.552 | 113.824, 381.280 | - |
|  |  |  |  |  |  |  | TC | -36.671 | -53.535, -19.808 | 1.709 |
|  |  |  |  |  |  |  | Age | -1.266 | -2.006, -0.527 | 1.015 |
|  |  |  |  |  |  |  | WC | 1.398 | 0.359, 2.436 | 1.054 |
|  |  |  |  |  |  |  | LDL-C | -20.077 | -36.110, -4.043 | 1.642 |

BMI: body mass index; LFC: liver fat content; DBP: diastolic blood pressure; TC: total cholesterol; ApoA1: apolipoprotein A1; WC: waist circumference; LDL-C: low-density lipoprotein cholesterol; VIF: variance inflation factor
